# Digital Subtraction Angiography of Cerebral Arteries: Influence of Cranial Dimensions on X-ray Tube Performance

**DOI:** 10.3390/jcm13103002

**Published:** 2024-05-20

**Authors:** Sandra Modlińska, Łukasz Czogalik, Marcin Rojek, Piotr Dudek, Michał Janik, Sylwia Mielcarska, Jakub Kufel

**Affiliations:** 1 Department of Radiodiagnostics, Invasive Radiology and Nuclear Medicine, Faculty of Medical Sciences in Katowice, Medical University of Silesia, 40-055 Katowice, Poland; 2 Department of Radiology and Nuclear Medicine, Faculty of Medical Sciences in Katowice, Medical University of Silesia, 40-055 Katowice, Poland; 3 Students’ Scientific Association of Computer Analysis and Artificial Intelligence, Department of Radiology and Nuclear Medicine, Faculty of Medical Sciences in Katowice, Medical University of Silesia, 40-055 Katowice, Poland; 4 Department of Medical and Molecular Biology, Faculty of Medical Sciences in Zabrze, Medical University of Silesia, 40-055 Katowice, Poland; 5 Department of Biophysics, Faculty of Medical Sciences in Zabrze, Medical University of Silesia, 40-055 Katowice, Poland

**Keywords:** cerebral aneurysms, digital subtraction angiography (DSA), radiographic image enhancement, X-ray machine parameters, diagnostic imaging, cranial dimensions, radiation exposure, gender differences, biplane imaging

## Abstract

**(1) Background**. Digital subtraction angiography (DSA) is indispensable for diagnosing cerebral aneurysms due to its superior imaging precision. However, optimizing X-ray parameters is crucial for accurate diagnosis, with X-ray tube settings significantly influencing image quality. Understanding the relationship between skull dimensions and X-ray parameters is pivotal for tailoring imaging protocols to individual patients. **(2) Methods**. A retrospective analysis of DSA data from a single center was conducted, involving 251 patients. Cephalometric measurements and statistical analyses were performed to assess correlations between skull dimensions and X-ray tube parameters (voltage and current). **(3) Results**. The study revealed significant correlations between skull dimensions and X-ray tube parameters, highlighting the importance of considering individual anatomical variations. Gender-based differences in X-ray parameters were observed, emphasizing the need for personalized imaging protocols. **(4) Conclusions**. Personalized approaches to DSA imaging, integrating individual anatomical variations and gender-specific differences, are essential for optimizing diagnostic outcomes. While this study provides valuable insights, further research across multiple centers and diverse imaging equipment is warranted to validate these findings.

## 1. Introduction

Brain aneurysms are a dangerous vascular pathology, affecting up to 6% of the population depending on the country [1]. They are usually asymptomatic, often diagnosed incidentally during unrelated medical investigations. This is why an effective and unambiguous diagnosis is crucial. Despite advancements in imaging technologies, there remains a need to better address the challenges associated with detecting intracranial aneurysms, particularly in understanding the intricacies of imaging techniques for this purpose [2]. Digital subtraction angiography (DSA) is the method that provides the highest-precision imaging of vascular pathology and thus achieves the highest sensitivity, according to recent studies [3]. In difficult and ambiguous situations, DSA maintains its position as the gold standard in the evaluation of cerebral aneurysms, demonstrating superior diagnostic performance compared to other available imaging techniques, such as angio-CT and angio-MR [4]. DSA imaging involves selectively catheterizing arterial vessels of the central nervous system, injecting an iodine contrast agent directly into the catheterized artery, and then capturing a series of X-rays before and after contrast administration. This enables the acquisition of both 2D and 3D images (due to rotation) of the blood vessels and any pathologies present [5]. Depending on the indication, the examination may involve only one side (right or left) or both sides of the patient. However, it is imperative to obtain images of the highest possible quality to ensure accurate diagnosis and treatment planning [6]. Direct factors affecting the quality of X-ray images include the X-ray tube voltage (kV), tube current (mA), and exposure time. The applied voltage on the X-ray tube influences the depth of penetration of the radiation beam—the higher the voltage, the greater the penetration of the patient’s body, resulting in more detailed images [7]. Commercially available C-arm X-ray systems are equipped with automatic exposure control (AEC) systems, designed to adjust exposure parameters such as kV, mA, and exposure time to the individual physique of the patient. AEC systems aim to ensure that the resulting images are of the highest possible quality by delivering sufficient X-ray photons to the detectors [8]. However, because DSA studies use ionizing radiation, they carry the risk of both stochastic and deterministic effects [9]. Given these considerations, the structure of the patient, specifically the size and thickness of the skull, should directly influence the parameters of the X-ray tube during DSA examinations. This is the aspect that we decided to investigate.

## 2. Materials and Methods

**Figure 1:**
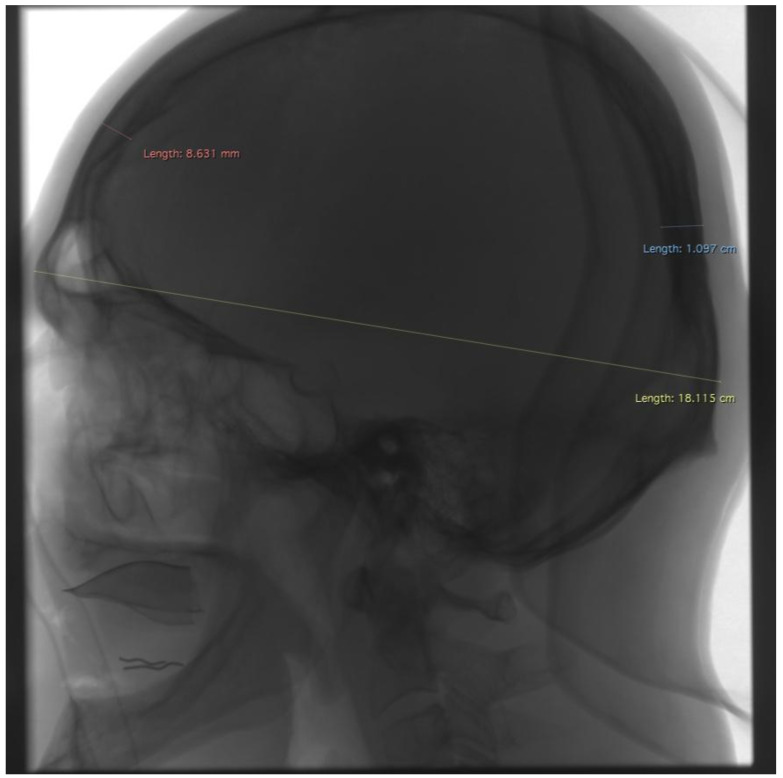
Example of measurements taken in lateral projection. Red—the greatest cranial thickness near the point g, blue—the greatest cranial thickness near the point op, yellow—the greatest cranial length (M1).

**Figure 2:**
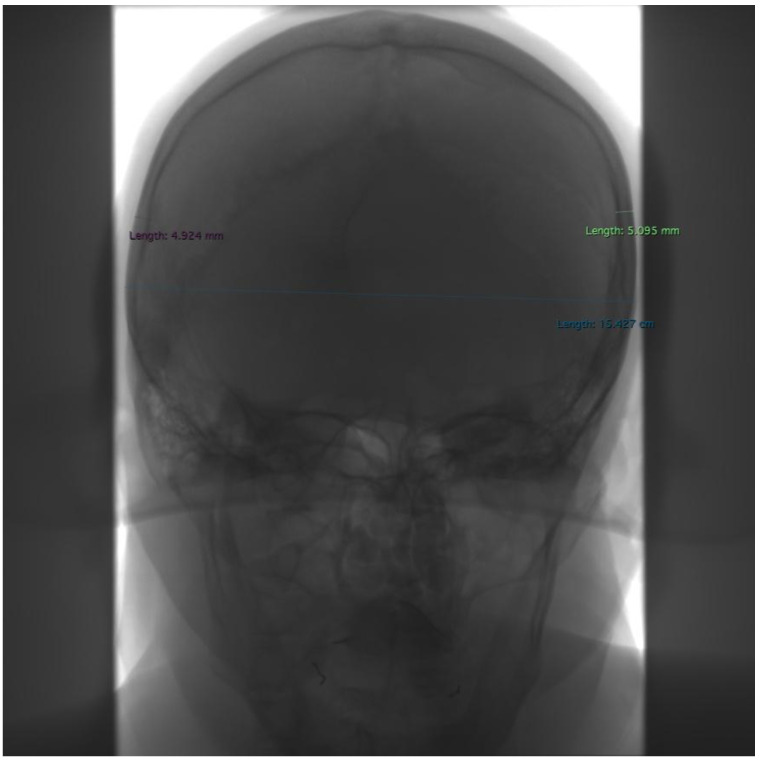
Example of measurements taken in frontal projection. Purple—the greatest cranial thickness near the right eu point, green—the greatest cranial thickness near the left eu point, turquoise—the widest cranial width (M8).

One center retrospectively analyzed medical records from a prospectively maintained database of patients who underwent DSA. All DSA examinations were performed between 31 January 2020 and 9 April 2021 in patients with suspected or confirmed central nervous system vascular aneurysms. Images were obtained using a GE Innova IGS 630 (GE HealthCare Technologies Inc., Chicago, IL, USA) C-arm biplanar angiosuite using the ARTERIOGRAPHY MO protocol. During the conducted examination, the following data were collected: gender, X-ray tube parameters (kV and mA), information about the rotation of the C-arm during the examination, the number of projections performed, information about the side of the examination (right/left/both sides), the presence or absence of metal coils (after a previous embolization procedure). Contrast injection rate, in our hospital, is typical for this exam. Value of the contrast is comparable in every data. This is the reason for why authors do not analyze these parameters. Using Horos software, cephalometric measurements were taken on radiographs in PA and lateral projections. Measurements included the widest cranial width (M8), the greatest cranial thickness near the points (eu, which is the beginning and end of the chord guided to delineate the M8 line) on both sides (Figure 1), the greatest cranial length (M1), and the greatest cranial thickness near the points (g and op, which are the beginning and end of the chord guided to delineate the M1 line) on both sides (Figure 2). A total of 251 patients (181 women, 70 men) were included. The number of unilateral DSA studies was 72 L and 64 P (136 in total). The number of bilateral DSA studies was 115. A total of 251 cephalometric measurements were taken, of which 6 parameters (M8, M1, 2× eu, op, and g) were measured in each study. The scheme of measurements is shown in Figure 1 and Figure 2. The collected data were subjected to statistical analysis using Statistica Statsoft V14.0 EN software and, in the Python environment, using the SciPy library, employing a series of statistical tests. For all analyses, the significance of results was assumed for *p* less than 0.05. Spearman’s rank correlation was used to analyze continuous data corresponding to successive dimensions (M8, eu, M1, g, and op) with voltage and current. The Mann–Whitney U test was used to analyze the correlation of nominal data (gender, coils) with X-ray tube parameters. Due to the presence of outliers, the dataset was cleaned by applying an interquartile cut-off to the analyzed variables. Thus, 27 records were excluded, leaving 224 measurements for the analysis of X-ray tube parameters.

## 3. Results

Of all patients, 27.89% were men, and 72.11% were women. The distribution of sides of DSA performed in men was 30% L, 21.43% P, and 48.57% L + P, while, in women, the distribution was 28.18% L, 27.07% P, and 44.75% L + P. A total of 28.69% of patients had DSA on the left side (L), 25.50% on the right side (P), and 45.82% on both sides (L + P). The mean value of kV was 71.06, and that of mA was 166.3, with women having values of 71.06 kV and 163.88 mA, and men having values of 72.93 kV and 172.56 mA. A coil was present in 127 studies, which accounts for 50.59 percent of all studies.

The mean values were as follows: M8 was 148.72 mm (152.58 for males, 147.23 for females), M1 was 176.68 mm (182.56 for males, 174.41 for females), the highest mean thickness near the eu point on the left side was 4.5 mm (4.9 for males, 4.65 for females), the highest mean thickness near the eu point on the right side was 4.57 mm (4.09 for males, 4.76 for females), the greatest mean thickness near the g point was 6.38 mm (7.28 for males, 6.98 for females), and the greatest mean thickness near the op point was 7.03 mm (7.28 for males, 6.93 for females).

**Figure 3:**
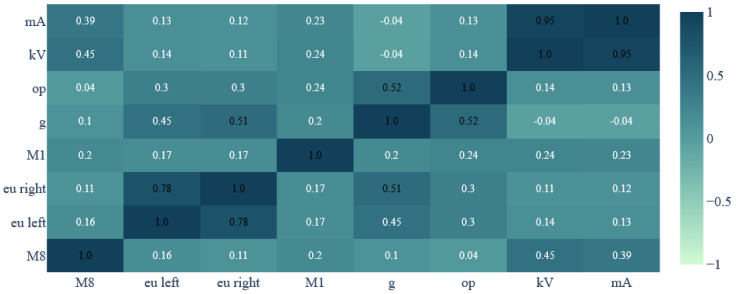
Heatmap showing correlation matrix between variables.

**Figure 4:**
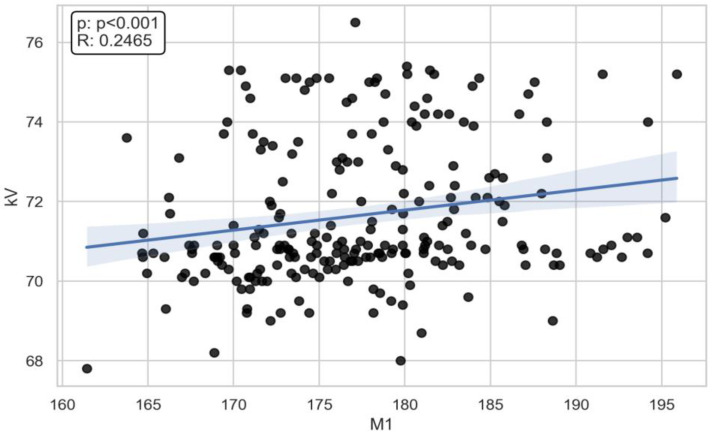
The Spearman correlation between M1 and voltage (R = 0.2465, *p* < 0.001).

**Figure 5:**
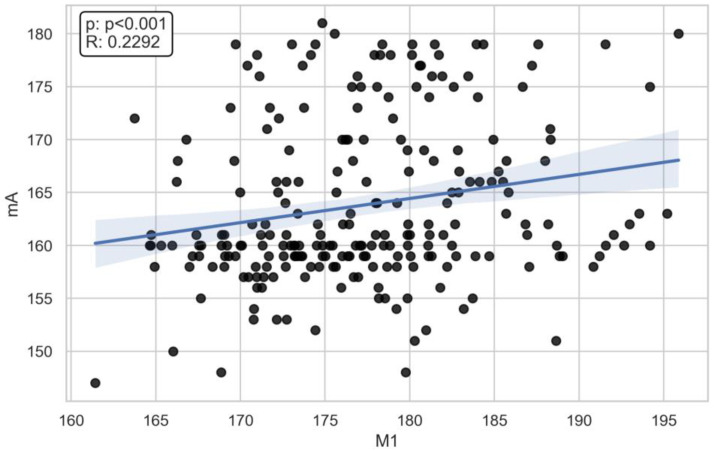
The Spearman correlation between M1 and current (R = 0.2292, *p* < 0.001).

**Figure 6:**
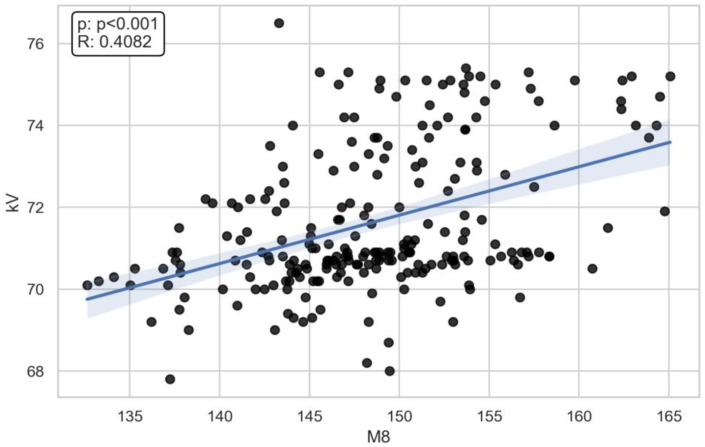
The Spearman correlation between M8 and voltage (R = 0.4082, *p* < 0.001).

**Figure 7:**
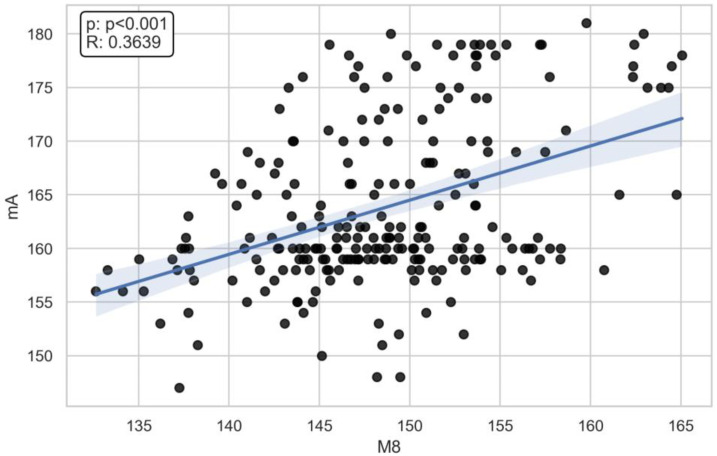
The Spearman correlation between M8 and current (R = 0.3639, *p* < 0.001).

**Figure 8:**
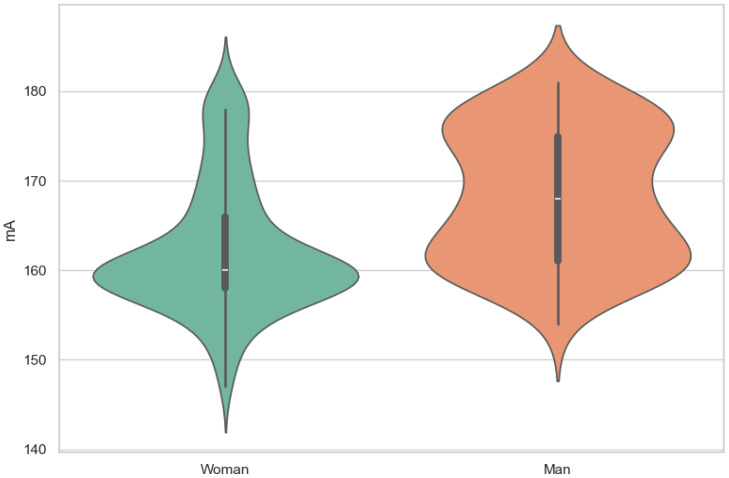
The violin plot of current by gender.

**Figure 9:**
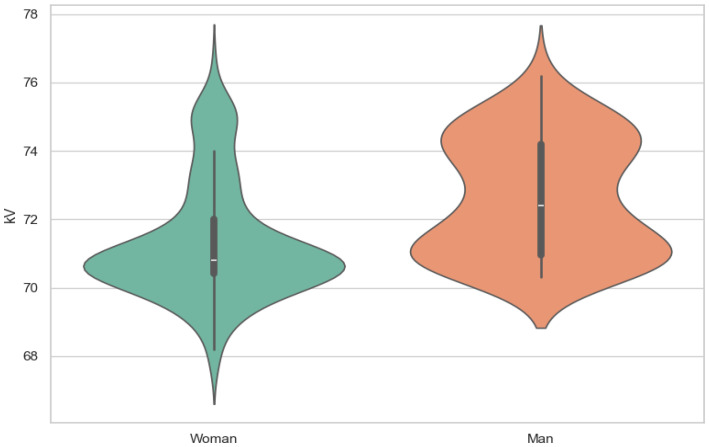
The violin plot of voltage by gender.

There was a moderate correlation between the M8 ($x^{-}$ = 148.7 mm) dimension and voltage (ρ = 0.45) ($x^{-}$ = 71.6 kV) and current (ρ = 0.39) ($x^{-}$ = 166 mA) (Figure 3, Figure 4, Figure 5, Figure 6 and Figure 7). The Spearman correlation between M8 and voltage or current is shown in Figure 6 and Figure 7. Additionally, a weak correlation was shown with the M1 dimension (for voltage, ρ = 0.24; for current, ρ = 0.23). The Spearman correlation between M1 ($x^{-}$ = 176.7 mm) and voltage or current is shown in Figure 4 and Figure 5. Bigger M1 gives higher parameters. *p* < 0.001 shows statistically significant differences between the obtained results for M1 and M8. Based on the Mann–Whitney U-test, there was a statistically significant association between gender and current (U: 2817, *p* < 0.001) and voltage (U: 2773, *p* < 0.001) (Figure 8 and Figure 9). There was no association between the presence of a coil and X-ray tube performance (*p* > 0.05, 0.73 for mA, and 0.14 for kV).

## 4. Discussion

In summary, this study revealed notable disparities between men and women concerning mean values of technical parameters, cranial dimensions, and thickness in various areas. Men exhibited higher values across these metrics compared to women, highlighting inherent anatomical differences between genders that can influence radiographic outcomes [10].

The observed moderate correlation between M8 dimensions and voltage/current, along with the weaker correlation between M1 dimensions and voltage/current, suggests that individual anatomical variations play a crucial role in determining optimal X-ray tube settings during DSA examinations. These findings underscore the necessity for personalized approaches to imaging, wherein adjustments to X-ray tube parameters should be tailored to accommodate differences in cranial anatomy.

Moreover, the statistically significant associations between gender and X-ray tube current/voltage emphasize the importance of considering patient gender when configuring X-ray tube parameters. The documented lower parameters in women indicate the need for nuanced adjustments to ensure adequate exposure and image quality, particularly in light of potential differences in cranial size and thickness between genders.

Interestingly, the lack of significant influence of a coil on X-ray tube parameters within the scope of this research suggests its minor importance in the context of DSA examinations. While a coil may play a role in other imaging modalities or specific clinical scenarios, its impact appears negligible in this particular study.

Overall, these findings underscore the complexity of optimizing X-ray parameters in DSA examinations and highlight the importance of considering individual anatomical variations, gender-specific differences, and other factors when configuring imaging protocols for optimal diagnostic outcomes.

It is worth noting the innovativeness of the problem undertaken in the study that we conducted. In the available literature, this is the first work of its kind on DSA. Researchers have so far addressed the topic of the influence of patient body size parameters on radiation exposure, mainly in CT scans.

One notable study by Huda et al. [11] stands out in the existing literature landscape. Their research aimed to elucidate the complex interplay between patient age, body size, composition, and X-ray technique parameters on radiation doses in head CT scans. In this groundbreaking study, Huda et al. [11] meticulously analyzed a cohort of 127 patients, assessing parameters such as head dimensions, cross-sectional area, and Hounsfield unit values on head CT images.

The dose calculations conducted by Huda et al. [11] encompassed cross-sections totaling 1837 mm, with scans performed at a consistent current of 340 mAs across varying X-ray tube voltages ranging from 80 to 140 kV. Their findings revealed compelling insights into the differential radiation absorption between adult men and women, with adult men exhibiting approximately 5% larger head dimensions and correspondingly higher radiation absorption rates.

These findings underscore the critical importance of accounting for patient-specific factors, including age, head size, composition, and X-ray tube parameters, when assessing radiation doses in CT imaging. By integrating these factors into dose calculations, healthcare professionals can ensure more accurate estimations of radiation exposure and tailor imaging protocols to optimize patient safety and diagnostic efficacy.

Overall, the pioneering work of Huda et al. and our own study highlights the necessity of a comprehensive understanding of the complex relationships between patient characteristics and radiation exposure in medical imaging. Moving forward, continued research efforts in this domain will be essential for refining imaging protocols, enhancing patient care, and mitigating potential risks associated with ionizing radiation exposure [11].

Israel et al. in their study investigated the effect of patient size on the amount of radiation used during CT scans of the chest, abdomen, and pelvis with automatic exposure control, as well as on the corresponding patient doses. The study’s findings underscore the need to take patient weight into account when assessing radiation doses in CT examinations with automatic exposure control [12].

It is worth noting that, in a study by Yi et al., which aimed to evaluate methods to reduce radiation exposure during routine DSA, analysis of factors affecting radiation dose showed that age and aneurysm diagnosis remarkably affected the radiation dose, while gender or other diagnostic factors showed no significant relationship. However, it should be added to the above conclusion that, apart from the aforementioned criteria, no other physical data of patients were taken into account (nor was this the main purpose of this study) [13].

If one were to shed some light on the biases and limitations in the methodology of our study, it was a retrospective analysis of data from a single center. This may limit the representativeness of the results for the general population. The specific local conditions and study period from January 2020 to April 2021 may introduce some bias due to potential changes in clinical practices, technology, or patient characteristics at different times. In addition, the use of specific equipment, i.e., the GE Innova IGS 630 biplanar angiosuite, may affect technical parameters. Despite the cephalometric analysis, not all potentially relevant anatomical parameters were included, which is also a methodological limitation. A retrospective study is also associated with a lack of control over external factors, such as variability in medical personnel or examination techniques [14]. Noting these limitations at the same time makes it possible to conduct analogous analyses by other institutions and compare them with the results that we obtained.

Future studies could be extended to multicenter, which would increase the representativeness of the results. Extending the study period would allow for a more comprehensive analysis of changes over time. It would also be worthwhile to include different models of biplanar angiosuites to assess the impact of equipment specificity on the results [15]. A more complete cephalometric analysis, taking into account additional anatomical parameters and the density of structures within the head, as well as the introduction of follow-up studies and randomization of patients, could also increase the value of the study. However, monitoring changes in medical technology and clinical practices will be key to adapting the study to the evolving medical context [16]. All of these solutions are aimed at making future studies more representative, objective, and adapted to the changing medical environment.

Last but not least, a significant relationship exists between X-ray tube parameters and the cranial dimensions of patients undergoing DSA examinations. This discovery implies that adjusting these parameters to individual patient characteristics could influence the quality of images obtained during DSA examinations. In clinical practice, considering these relationships may be essential, enhancing diagnostic accuracy and minimizing patient exposure to X-rays. All angiosuites, as well as digital radiology, use AEC (automatic exposure control) systems. This means that the X-ray tube uses automatic parameters depending on the patient’s anatomical parameters. Manual modification of the protocol may mean that each hospital will have different protocols and test parameters, regardless of the patient’s parameters, which is not recommended. It is also worth remembering that manually changing the operating parameters of the X-ray tube may negatively affect the quality of the obtained images; therefore, it is recommended to use AEC systems. Nevertheless, diagnostic accuracy and image quality are not only from X-ray tube parameters. Just like images obtained from CT or MRI machines, images obtained from DSA are also viewed and evaluated at diagnostic stations. Each station is equipped with software that allows one to change the contrast and brightness of the image. However, it is worth remembering that any image manipulation may result in the loss of diagnostic information. It is worth noting that there are no standards that specify what images are considered diagnostic. It is also worth mentioning that monitors and their technical condition also have a huge impact on the quality of the assessed images.

The amount of contrast administered to individual patients was not analyzed, as our center uses standardized volumes of contrast administration that are the same for a given projection; when performing an acquisition for each patient, the value of contrast flow per second, the number of images per second (frames) is set, so these values can be standardized for each patient. In addition, agents with the same concentration of iodine are used, which translates into consistent X-ray tube performance.

This standardized approach to contrast administration reflects established protocols at our institution, designed to optimize image quality and procedure performance while maintaining patient safety. By maintaining a consistent injection rate and volume of contrast, healthcare professionals can minimize variability in contrast enhancement during DSA examinations, thereby facilitating a more accurate interpretation of vascular anatomy and pathology.

Although contrast injection rate and volume are key factors in DSA imaging, their homogeneity in the datasets at our hospital limits the need for detailed analysis in this study. Instead, we focused on other key variables, such as X-ray tube parameters and patient anatomical features, which are more directly influenced by differences in imaging protocols and patient demographics.

Overall, the standardized approach to contrast administration underlines our commitment to providing quality care and optimizing imaging outcomes for patients undergoing DSA. By adhering to established protocols and continuously monitoring procedural parameters, we aim to provide consistent and reliable results across all imaging studies. The authors would like to emphasize that working with the use of AEC systems makes it possible to obtain good diagnostic images without the need to experiment with X-ray tube parameters. However, it is worth remembering that, in the case of patients with larger skull dimensions, limiting the number of images taken will directly affect the dose received by the patient. Nevertheless, showing angiosuite manufacturers the relationship between skull size and X-ray tube operating parameters may contribute to improving the protocols used in DSA research.

## 5. Conclusions

Following the analysis of skull measurements and their correlations with X-ray tube parameters (voltage—kV, current—mA), several significant insights emerge. Firstly, the moderate correlation observed between the M8 dimension and voltage (ρ = 0.45) and current (ρ = 0.39) highlights the significance of these parameters in determining image quality and diagnostic accuracy during DSA examinations. Similarly, the weak correlation between M1 and tube parameters (for voltage ρ = 0.24; for current ρ = 0.23) underscores the complexity of the relationship between skull dimensions and X-ray settings.

These findings underscore the critical importance of considering both M8 and M1 dimensions when conducting DSA examinations, particularly in the commonly utilized PA and lateral projections. Given the fundamental principles of X-ray physics, adjustments to the voltage directly impact the wavelength and depth of penetration of the X-ray beam. Consequently, selecting appropriate kV values becomes pivotal in ensuring optimal image acquisition, especially when dealing with variations in tissue density or thickness across different anatomical regions.

Furthermore, the observed relationship between gender and X-ray tube parameters, with women exhibiting lower X-ray tube current (for current *p* < 0.001) and voltage (*p* < 0.001), sheds light on potential gender-based differences in cranial anatomy. The documented variations in cranial size and thickness between genders emphasize the need for tailored imaging protocols to account for individual anatomical variations. Notably, the implications of these findings extend beyond gender alone, as individuals with larger heads or thicker skull bones may also warrant special consideration to mitigate the risk of overexposure to ionizing radiation.

However, it is crucial to acknowledge the limitations inherent in this study. The data were collected from a single center using a specific X-ray device (GE Innova IGS 630), which may limit the generalizability of the findings. To ensure broader applicability, future research should encompass multiple centers and diverse imaging equipment to validate these conclusions on a more comprehensive scale.

## Data Availability

The original contributions presented in the study are included in the article; further inquiries can be directed to the corresponding author.
